# Analysis of Recurrent Times-to-Clinical Malaria Episodes and *Plasmodium falciparum* Parasitemia: A Joint Modeling Approach Applied to a Cohort Data

**DOI:** 10.3389/fepid.2022.924783

**Published:** 2022-07-08

**Authors:** Christopher C. Stanley, Mavuto Mukaka, Lawrence N. Kazembe, Andrea G. Buchwald, Don P. Mathanga, Miriam K. Laufer, Tobias F. Chirwa

**Affiliations:** ^1^Faculty of Health Sciences, School of Public Health, University of the Witwatersrand, Johannesburg, South Africa; ^2^Malaria Alert Center, Kamuzu University of Health Sciences, Blantyre, Malawi; ^3^Oxford Centre for Tropical Medicine and Global Health, Oxford, United Kingdom; ^4^Mahidol-Oxford Tropical Medicine Research Unit, Bangkok, Thailand; ^5^Department of Statistics, University of Namibia, Windhoek, Namibia; ^6^Center for Vaccine Development and Global Health, University of Maryland School of Medicine, Baltimore, MD, United States

**Keywords:** *Plasmodium falciparum*, longitudinal data, malaria parasitemia, recurrent clinical malaria, cox proportional hazards model, mixed-effects model, shared frailty model, joint modeling

## Abstract

**Background:**

Recurrent clinical malaria episodes due to *Plasmodium falciparum* parasite infection are common in endemic regions. With each infection, acquired immunity develops, making subsequent disease episodes less likely. To capture the effect of acquired immunity to malaria, it may be necessary to model recurrent clinical disease episodes jointly with *P. falciparum* parasitemia data. A joint model of longitudinal parasitemia and time-to-first clinical malaria episode (single-event joint model) may be inaccurate because acquired immunity is lost when subsequent episodes are excluded. This study's informativeness assessed whether joint modeling of recurrent clinical malaria episodes and parasitemia is more accurate than a single-event joint model where the subsequent episodes are ignored.

**Methods:**

The single event joint model comprised Cox Proportional Hazards (PH) sub-model for time-to-first clinical malaria episode and Negative Binomial (NB) mixed-effects sub-model for the longitudinal parasitemia. The recurrent events joint model extends the survival sub-model to a Gamma shared frailty model to include all recurrent clinical episodes. The models were applied to cohort data from Malawi. Simulations were also conducted to assess the performance of the model under different conditions.

**Results:**

The recurrent events joint model, which yielded higher hazard ratios of clinical malaria, was more precise and in most cases produced smaller standard errors than the single-event joint model; hazard ratio (HR) = 1.42, [95% confidence interval [CI]: 1.22, 2.03] vs. HR = 1.29, [95% CI:1.60, 2.45] among participants who reported not to use LLINs every night compared to those who used the nets every night; HR = 0.96, [ 95% CI: 0.94, 0.98] vs. HR = 0.81, [95% CI: 0.75, 0.88] for each 1-year increase in participants' age; and HR = 1.36, [95% CI: 1.05, 1.75] vs. HR = 1.10, [95% CI: 0.83, 4.11] for observations during the rainy season compared to the dry season.

**Conclusion:**

The recurrent events joint model in this study provides a way of estimating the risk of recurrent clinical malaria in a cohort where the effect of immunity on malaria disease acquired due to *P. falciparum* parasitemia with aging is captured. The simulation study has shown that if correctly specified, the recurrent events joint model can give risk estimates with low bias.

## Introduction

Joint modeling of longitudinal and time-to-event data has recently received increased attention in biomedical research (1–3). Typically, a joint model consists of two parts: a model for time-to-event process; and a model for the longitudinal process. The joint modeling approach is gaining popularity (1–4), partly because it offers the advantage of capturing important relationships between longitudinal outcomes and time-to-event processes that are otherwise lost by separate longitudinal and survival analyses (1, 5). However, joint models that can handle recurrent events are limited. Recent methodological and software developments in joint modeling have been extensively reviewed elsewhere (1–3, 6–8). Applications in the reviewed studies of joint modeling have typically focused on time-to-single event only. For example, studies have frequently modeled longitudinal CD4 count jointly with time-to-HIV–related outcomes in order to understand the relationships between the longitudinal history of CD4 and its effect on the risk of development of AIDS (1, 9–11). Among patients with cancer, repeated measurements of quality of life performance scores have been jointly modeled with time-to-death or disease progression (12–15). However, for diseases that may have multiple episodes such as clinical malaria, chronic heart failure, epileptic seizures, or asthma attacks, modeling that focuses on time-to-first event only while ignoring subsequent events may not be efficient since such approaches fail to utilize all information available in the data (6, 16, 17). In malaria, single-event models do not capture the role of acquired immunity, which develops with repeated *Plasmodium falciparum* infections over time, to future disease episodes (18). The WHO Malaria Vaccine Advisory Committee (MALVAC) has recently recommended analyzing recurrent event times to evaluate malaria vaccines (19).

In *P. falciparum* malaria studies, modeling of time-to-single malaria episode may not be accurate especially in malaria-endemic regions because recurrent clinical disease is frequently observed. Instead, modeling the risk of disease including all the recurrent events during follow up may provide improved accuracy. Repeated infection is common and with each infection, acquired immunity develops making subsequent disease and infection episodes less likely (20, 21). Therefore, modeling recurrent clinical disease episodes jointly with the longitudinal measurements of *P. falciparum* parasitemia data may be critical to capturing the effect of the developing immunity to malaria.

The joint model of recurrent events and a longitudinal outcome typically consists of a recurrent events model and a mixed-effects model linked through either latent variables (22, 23) or shared random effects (17, 24). The most common approach used to model the recurrent events process is the shared frailty model introduced by Clayton (25, 26), which usually takes the Gamma distribution. For the longitudinal process, studies have frequently focused on continuous (Gaussian) outcomes and often applied linear mixed-effects models (5, 17, 27). However, the use of linear mixed-effects models for longitudinal outcomes is not appropriate for outcomes with Poisson or NB distributions. Joint modeling of recurrent events and a non-Gaussian longitudinal outcome such as the *P. falciparum* parasitemia remains a challenge.

In this study, a joint model is proposed which comprised a shared frailty model for recurrent malaria episodes and an NB mixed-effects model for repeated measurements of *P. falciparum* parasitemia. The proposed approach was motivated by data from a prospective malaria cohort in Malawi, which has been described previously (28–31). Malaria is endemic in Malawi (32) and transmission of the *P. falciparum* parasite is high in the area of the study (33, 34). We used data from a clinical study to investigate whether jointly modeling time-to-recurrent clinical malaria episodes with longitudinal parasitemia may yield more accurate risk factor estimates compared to a single-event joint model (for time-to-first clinical malaria episode and NB mixed-effects sub-model for the longitudinal parasitemia) where the subsequent episodes are ignored. Here, aging of participants was considered as a proxy for increasing levels of acquired immunity. The recurrent events joint model is also tested for the prediction of a new clinical malaria episode given the history of recurrent events and *P. falciparum* parasitemia trajectory. Finally, simulations were conducted to study the performance of the joint model under different conditions.

## Materials and Methods

### Data Source

The joint models were applied to data from the prospective cohort study conducted in a rural area of southern Malawi. The cohort enrolled 120 participants who presented with uncomplicated malaria at a rural health center between June 2014 and March 2015. The study design was described previously (29). Study participants were actively followed monthly and on interim malaria sick visits for up to 2 years post enrolment.

### Outcomes

The primary outcomes of the study were recurrent clinical disease defined as participants' self-reported fever and with a positive rapid diagnostic test (RDT) result; and density of *P. falciparum* infection: parasitemia was measured as the number of parasites per microliter (μl) of blood. Parasitemia measurements were obtained from thick blood smears.

### Covariates

The models included participants' age as continuous, gender, the season of the previous visit categorized as dry (May–November) or rainy (December–April), and use of long-lasting insecticide-treated bed net (LLIN) in the previous month before the visit dichotomized as whether one reported using the LLINs every night or not.

### Data Structure

A sample of the data structure showing three hypothetical participants for the analysis of time-to-recurrent clinical malaria episodes is provided in Supplementary Table 1. The time of origin for the analysis of the recurrent episodes was the day of study enrolment. A clinical disease episode was considered new if it occurred >14 days after the previous episode based on the pharmacokinetics of artemether–lumefantrine treatment in the study (35).

### Notation and Specification of Models

#### The Longitudinal Sub-model

In the longitudinal setting, let *Y*_*ij*_ denote the longitudinal response of *P. falciparum* parasitemia for subject *i* = 1, …, *n* at time *j* where *j* = 1, …, *J*_*i*_. The measurements can be summarized as:


(1) $$\begin{array}{r} Y_{ij}=\mu_{i}+\psi_{i}+\epsilon_{ij}, \end{array}$$

where *μ*_*i*_ is the mean response of parasitemia, *ψ*_*i*_ are subject-specific random effects accounting for within-subject correlation in each model part, and *ϵ*_*ij*_ represent error terms and are assumed to be normally distributed, that is, $\epsilon_{ij}\sim N_{n_{i}}\left(0,\sigma^{2}I_{n_{i}}\right)\;$where σ^2^ is variance and *I*_*n*_*i*__ is the *n*_*i*_× *n*_*i*_ identity matrix. Postulating a model formulation proposed by Henderson et al. (5), assuming that *μ*_*i*_ can be described by a linear mixed-effects (LMEM) sub-model with a Gaussian distribution:


(2) $$\mu_{i}=\beta X_{i}^{'}+bZ_{i}^{'}+\epsilon_{i},$$

where *β* is the *p* × 1 fixed-effect parameter vector for the fixed-effect covariate vector *X*_*i*_, *b* is the *q* × 1 vector of random effects for random-effect covariate vector *Z* for participant *i*, assumed to be multivariate normal with mean zero, that is, $b_{i}\sim N_{q}\left(0,\underset{b}{\sum }\right)$, and $\underset{b}{\sum }is$ the variance of the subject-specific effects.

Taking NB distribution for the parasitemia, then


(3) $$\begin{array}{r} \left(y_{i}|\mu_{i},\vartheta \right)=\frac{\text{Γ}\left(y_{i}+\vartheta \right)}{\text{Γ}\left(\vartheta \right)y_{i}!}{.\;\left(\frac{\vartheta }{\mu_{i}+\vartheta }\right)}^{\vartheta }.\;{\left(\frac{\mu_{i}}{\mu_{i}+\vartheta }\right)}^{y_{i}}, \end{array}$$

where *μ*_*i*_ is the mean and ϑ is the shape parameter that accounts for over-dispersion. Parasitemia count data were tested for over-dispersion and considered the Negative Binomial (NB) model.

The NB mixed-effects model links the mean of response to the set of covariates through the logarithm function expressed as:


(4) $$\log\left(\mu_{i}\right)=\text{β}X_{i}^{'}+b_{i}Z_{i}^{'}+\log(M_{i}),$$

where log(*M*_*i*_) is the offset correcting for variation in the number of parasitemia measurements for subject *i*.

The NB distribution can be viewed as a Gamma mixture of Poisson distribution where the parasitemia response *y*_*i*_ with mean *μ*_*i*_ follows Poisson and subject-specific random effects error term *ψ*_*i*_ following the Gamma distribution. When the over-dispersion parameter is high, the NB model converges to a Poisson model and cannot deal with the over-dispersion (36), and is prone to non-convergence problems.

#### The Intensity Recurrent Event Sub-model

The recurrent event model extends the single event semi-parametric proportional hazards model by introducing an unobservable (frailty) random term on which the hazard function depends to account for within-participant dependence of events (37), that is, recurrent clinical malaria episodes. The single-event semi-parametric proportional hazards model can be expressed in terms of the hazard function λ_*i*_(*t*) for participant *i* as


(5) $${\text{λ}}_{i}(t)=\;{\text{λ}}_{0}(t)\exp\left[\beta X_{i}^{'}\right],$$

where λ_0_(*t*) is the unspecified baseline hazard function and *X*_*i*_ is the covariate vector for participant *i*. For ordinary Cox PH regression, the baseline hazard is usually left unspecified and can offer valid statistical inference using partial likelihood. However, in the context of joint modeling, a completely unspecified baseline hazard will generally lead to underestimation of the standard errors of the model parameters (8, 38). For recurrent clinical malaria episodes, an intensity event model function is adopted as opposed to a rate function because, while the rate function only defines the occurrence of recurrent events unconditional on the event history, the intensity function conditions the occurrence of events on the event history (39). In the case of recurrent malaria, the event history is particularly critical because each *P. falciparum* infection alters the host immune response against the threat of subsequent infections and disease episodes (20, 21). Thus, the intensity recurrent event model would account for the participants' strengthening immunity to clinical episodes due to accumulating event occurrences over time, which is critical in recurrent event analysis (23). The intensity recurrent event model at time *t* is given by the multiplicative intensity model following the structure proposed by Henderson et al. (5) as follows:


(6) $${\text{λ}}_{i}(t)=\;G_{i}{\text{λ}}_{0}(t)\exp\left[\beta X_{i}^{'}+\gamma_{i}\right],$$

where *G*_*i*_ is assumed to follow Bernoulli distribution denoting whether the participant *i* is in the risk period of experiencing the malaria episode. As with the single event survival model in Equation (5), the baseline hazard (intensity) function λ_0_(*t*) is assumed to follow Weibull distribution. In the current cohort data, the vectors *β* and *X*_*i*_ contain different sets of elements from α and *Z*_*i*_, respectively, in Equation (2). The term *γ*_*i*_ represents the unobservable random effects (frailty) term to account for dependence between within-participant episodes and is assumed to follow a Gamma distribution with unit mean and variance θ, i.e., *γ*_*i*_ ~ Γ(1, *θ*). The frailty variance θ, reflects the amount of the within-participant dependence of clinical episode times, that is, the correlation of the recurrent events is quantified by θ, with higher values corresponding to greater within-participant correlation. When the variance is small, the values of the frailty are around one, implying no frailty effects and so recurrent events are independent.

For the counting process of the recurrent clinical episodes, let $R_{i}^{*}\left(t\right)\;$be the number of recurrent events for subject *i* occurring before or at time *t* over an interval [0, *τ*], where recurrent episodes could potentially be observed beyond the prespecified maximum time point *τ*. Then the counting process may be stopped by the time of loss to follow-up or end of the study denoted by *C*_*i*_, with the failure indicator _*i*_ taking value 1 if *T*_*i*_ < *C*_*i*_ and 0 otherwise. The observed counting process, ${R_{i}=R}_{i}^{*}\min\left(t,C_{i}\right)$ has a known zero-one process {*G*_*i*_(*u*): 0 ≤ *u* ≤ *τ*} indicating whether the participant *i* is at risk of experiencing an episode during period *u*. Thus, the counting process $R_{i}^{*}\;$ has a jump of size one (*R, R* + 1, …) when an event occurs, that is, the episode of clinical malaria is experienced.

#### Likelihood of the Joint Model

Using generic terms *Y* to denote combined observed measurements of parasitemia data, *R* for combined recurrent episodes data, *X* for covariate information, and Φ = {*ψ*, *γ*} for the random and frailty processes, the joint distribution for the longitudinal measurements *Y* and recurrent event processes *R* are conditionally independent given *X*, *ψ*, and *γ*. The dependence between *Y* and *R* may arise from the direct link between *ψ* and *γ*, called latent association, without which nothing can be gained through a joint analysis. Our interest is to model the subjects' recurrent processes of episodes together with their longitudinal measurements of parasitemia, through the association of *ψ* and *γ*. Following the framework proposed by Henderson et al. (5), one can proceed to compute the likelihood of the joint model as a product of the marginal distribution of observed parasitemia measurements *Y* and the conditional distribution of the events (malaria episodes) *R*. For each participant *i*, the observed data are {(*t*_*i*_, *X*_*i*_, *u*_*i*_, Δ_*i*_, *τ*_*i*_), *i* = 1, 2, …, *n*}. Computing the full joint likelihood *L* = *L*(*π*, *Y, R*) where π = (*β*, *σ*, *θ*, λ_0_, *α*, Φ) is a vector denoting a collection of all unknown parameters with λ_0_ = {λ_0_ (*t*_*i*_), *i* = 1, 2, …, *n*}, one can proceed as follows:


(7) $$\begin{array}{r} L=L_{Y}\times L_{R|Y}=L_{Y}\left(\pi ,Y\right)\times E_{\psi |Y}\left\{L_{R|\gamma }\left(\pi ,R|\gamma \right)\right\}, \end{array}$$

here *L*_*Y*_(π, *Y*) is the standard form of the marginal distribution of *Y* for the parasitemia measurements process. The conditional likelihood of the recurrent episodes data, *L*_*R*|*γ*_(π, *R*|*γ*) captures the likelihood contribution of the longitudinal measurements up to any time of the event. Suppose we denote $R_{0}=\overset{t}{\underset{0}{\int }}\lambda_{0}\left(u\right)du$ as cumulative baseline intensity for the recurrent event process, then *L*_*R*|*γ*_(π, *R*|*γ*) can be expressed as


(8) $$\begin{array}{l} L_{R|\gamma }\left(\pi ,R|\gamma \right)=\prod_{t}\{\left({\prod_{t}\left[\exp\left\{\beta X_{i}^{'}+\gamma_{i}\right\}{\text{λ}}_{0}\right]}^{R_{i}}\right)\\ \;\times \;\exp\left(-\int_{0}^{\tau }G_{i}\exp\left\{\beta X_{i}^{'}+\gamma_{i}\right\}\right)dR_{0}\} \end{array}$$

The Gamma distribution of the frailty *γ* with mean restricted to 1 and variance θ, that is, *γ* ~ Γ(1, θ), can be expressed as $g\left(\gamma \right)=\frac{\theta^{1}\gamma^{1-1}e^{-\theta y}}{\Gamma \left(1\right)}$, *γ*_*i*_*ϵ*{0, ∞} which reduces to $g\left(\gamma_{i}\right)=\frac{\theta e^{-\theta y}}{\Gamma \left(1\right)}$.

#### Parameter Estimation

Estimates of the parameters are obtained by maximizing the joint likelihood for the parasitemia process and the recurrent episode times process using the EM algorithm. Estimating the parameters by maximizing the likelihood of the observed data involves integrating over the random and frailty terms, *γ*. Since the joint likelihood contains an analytically intractable integral, numerical methods of integration such as Bayesian approaches or quadrature approximation techniques are required for evaluation; we used the Gauss–Hermite quadrature method. Furthermore, a unit mean for the frailty term was assumed to make the parameters in the distribution and the baseline distribution λ_0_ identifiable.

The proposed joint modeling approach was applied to malaria cohort data from Malawi, as described above. Simulations were conducted to study how the joint model can perform under different conditions. The models were fitted with a shared Gamma frailty model for the recurrent events and a mixed-effects model taking competing distributions for the longitudinal process: Gaussian and NB. Model fit was compared based on the Akaike information criterion (AIC). The model with the lowest value of AIC was selected as the best-fitting model. Data were simulated to resemble the Mfera cohort. Age in years was assumed to be normally distributed (mean: 2, Standard Deviation [SD]: 0.8) on the log-scale. To maintain the skewness of age that would reflect real data, the simulated log-normally distributed values were transformed back to original scales by taking an exponential function. The covariates' gender, season, and LLIN usage were assumed to be binomially distributed. Based on exploratory analyses of the Mfera cohort, the assumed log of hazard values were −0.04 for age, 0.02 for gender, 0.3 for season, and 0.3 for LLIN usage. The baseline hazard function was assumed to follow a Weibull distribution with shape parameter lambda = 1 and scale parameter = 2. Follow-up time to event or censoring followed a uniform distribution. After each clinical malaria episode, a subject was assumed to be malaria-risk-free for 14 days, based on the pharmacokinetics of artemether–lumefantrine therapy. Parasitemia data measurements were simulated from a mixed-effects model with the function of follow-up time. The model bias was assessed under different scenarios that include study sample sizes of 100, 200, and 400 (representing small, medium, and large sample sizes); level of censoring 10, 20, and 50%; length of follow-up period of 1, 2, and 4 years; Gamma distributed frailty term with variances 0.2 for low dependence of within-participant episodes, 1.5 for moderate and 2.5 for highly dependent episodes; and correlation level between longitudinal and recurrent processes 0.01 for a weak association, 0.5 moderate, and 0.8 strong association. We hypothesized that the performance of the model would improve with increased study sample size, longer follow-up time, and strong association between the two processes, but that the performance would worsen with an increasing level of censoring. Simulations were conducted in R version 3.4.3 using package *simrec* (40). Data analysis was done in Stata SE version 15.1 (Stata Corp., College Station, TX, United States) using *gsem* and user-written program *merlin* (41).

## Results

### Malaria Cohort Study

**Figure 1:**
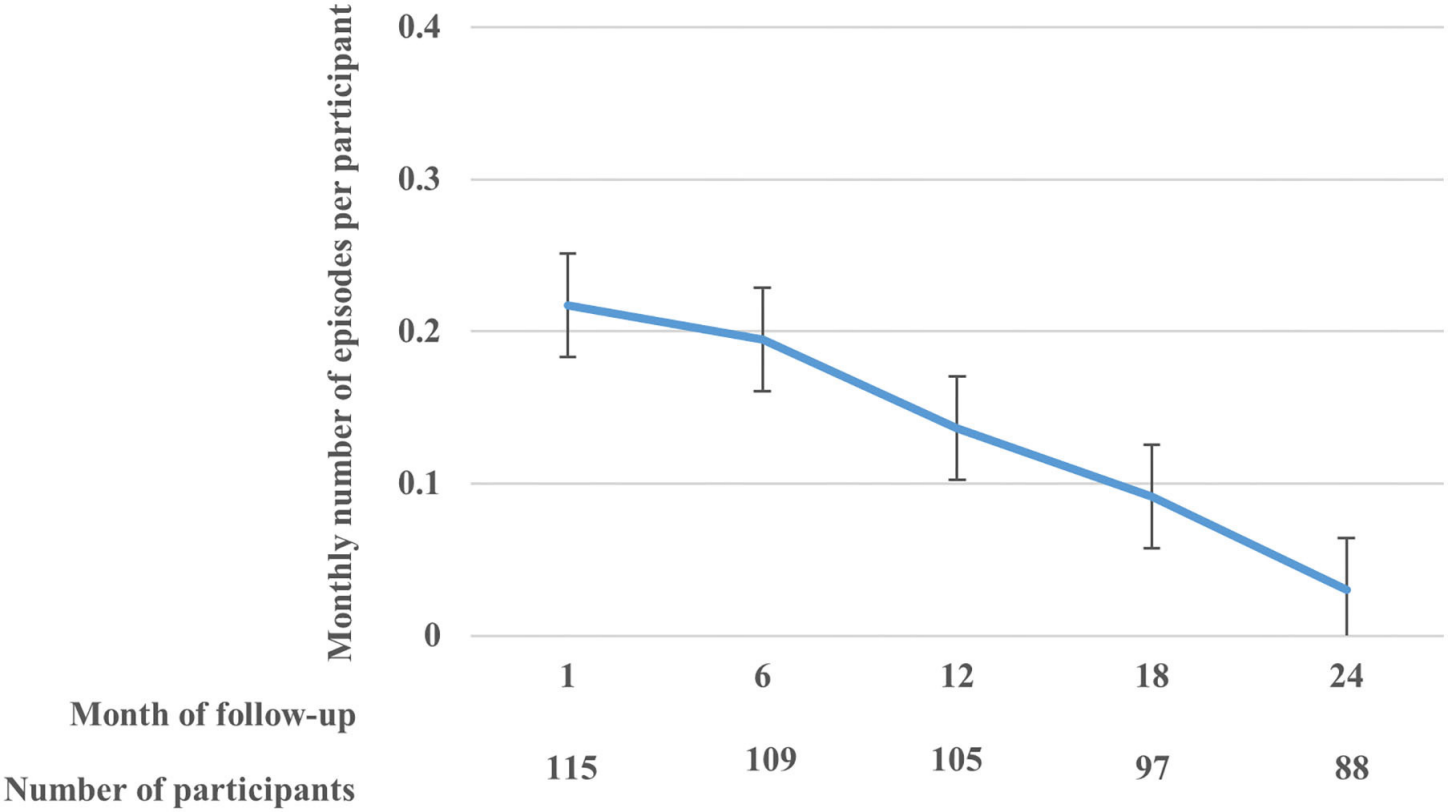
The rate (with standard error bars) of monthly clinical malaria episodes per participant over the follow-up period.

There were 120 participants in the cohort, of whom 69 (57.5%) were females. The overall median age was 7.5 years [inter-quartile range (IQR): 4.7–18.1]. The median number of malarial parasites per microliter was 11,060 (IQR: 840–54,000) overall, 24,840 (IQR: 1,600–68,600) in males, and 5,640 (IQR: 520–540,000) for females (Supplementary Table 2). The current analyses included data for 115 participants who had at least one follow-up visit post enrolment. Participants had a median of 37 visits (IQR: 29–45). There were 397 asymptomatic and 390 symptomatic cases in the cohort. Among these 115 participants, 372 recurrent clinical malaria episodes were experienced over the 2-year follow-up period, with a median of 3 episodes per person (IQR: 1–5). Overall, there was a decreasing rate of monthly recurrent clinical malaria episodes per participant over follow-up (Figure 1). Overall, the median level of parasitemia in the cohort was 24,400 parasites per microlitre (*μ*l) (interquartile range [IQR]: 1,240–76,700/*μ*l) during the follow-up period.

Hazard ratios for recurrent clinical malaria episodes obtained from the joint model of clinical malaria episodes and parasitemia are summarized in Table 1. The hazard of recurrent clinical malaria decreased with increasing participants' age HR = 0.96 [95% CI: 0.94, 0.98], for 1-year increase in age. The hazard of recurrent clinical malaria was higher among participants who reported not to use LLINs every night compared to those who reported using nets every night HR = 1.42, [95% CI: 1.22, 2.03]. Compared to observations in the dry season, the hazard of recurrent clinical malaria episodes was higher during the rainy season HR = 1.36, [95% CI: 1.05, 1.75]. The recurrent event joint model (left panel) yielded higher hazard ratio estimates of clinical malaria, which were more precise and in most cases with smaller standard errors, except for age compared to results from the single-event joint model (right panel).

**Table 1:** Hazard ratio (HR) estimates of clinical malaria among participants of Mfera cohort comparing recurrent events joint model vs. single-event joint model.

	**Recurrent events joint model**			**Single event joint model**		
**Variable**	**HR**	**SE**	**95% CI**	**HR**	**SE**	**95% CI**
Age, per year increase	0.96	0.01	0.94, 0.98	0.81	0.02	0.75, 0.88
Gender, female	1.18	0.13	0.96, 1.47	1.07	0.24	0.68, 1.70
Season, rainy^*^	1.36	0.21	1.05, 1.75	1.10	0.41	0.83, 4.11
Less LLIN use^+^	1.42	0.32	1.22, 2.03	1.29	0.33	1.60, 2.45

* *Rainy season (December–April), reference category is dry season (May–November)*.

+ *Reference category is LLIN use nightly in previous month*.

*HR, hazard ratio; SE, standard error; CI, confidence interval*.

**Figure 2:**
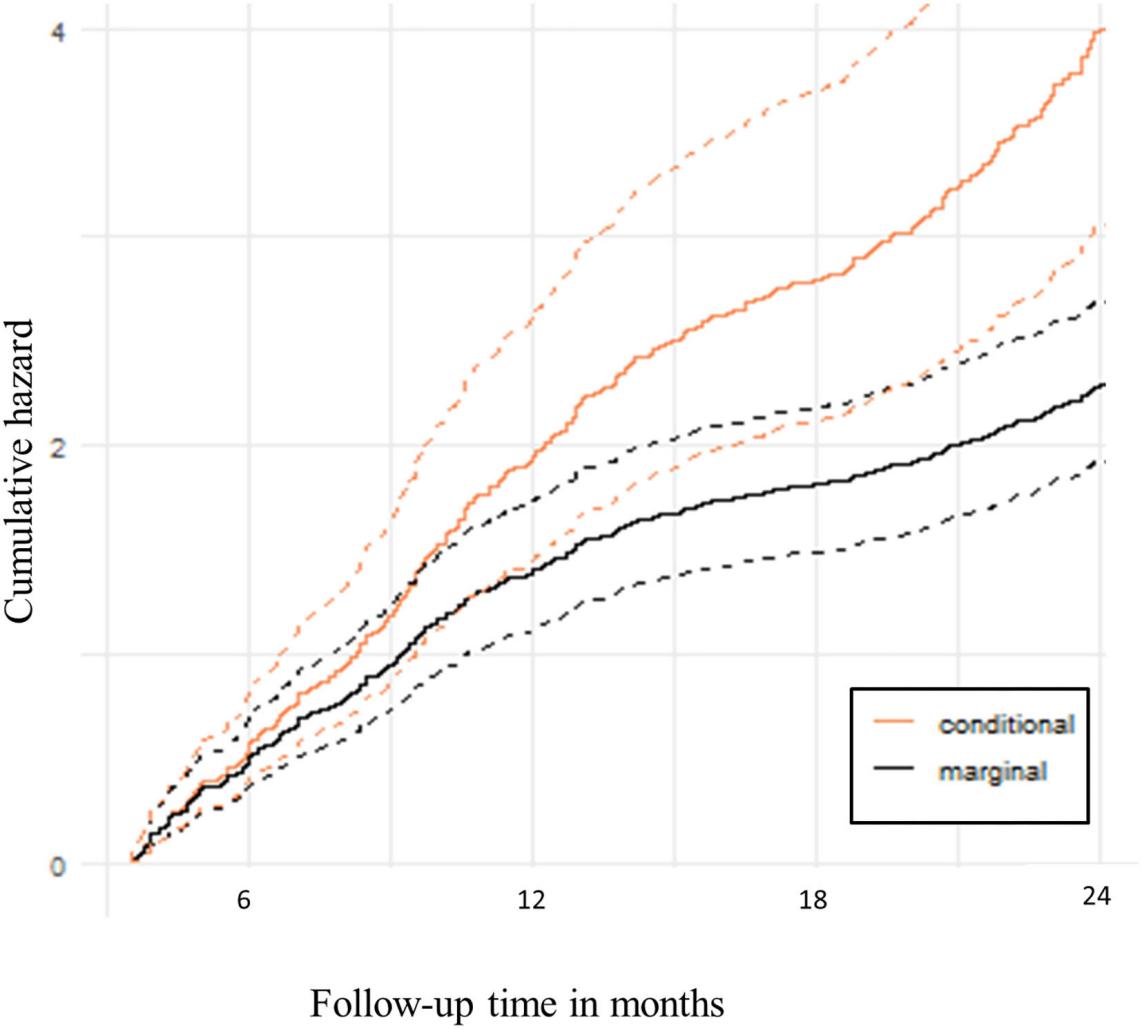
Predicted hazard of recurrent clinical malaria. The solid lines are cumulative hazards that represent the expected number of clinical malaria episodes over follow-up time. The dotted lines are the upper and lower bounds of the 95% Confidence bands.

The predicted conditional cumulative and marginal non-proportional hazards using the recurrent events joint model are shown in Figure 2. The expected number of clinical malaria episodes in the cohort increased sharply at the beginning of the follow-up period but later slowed down beyond 1 year. This shows that there were fewer clinical malaria episodes in subsequent periods over time.

### Simulation Study

Because the results from the recurrent events joint model presented in this study are based on data from a cohort of 115 participants with 2 years of follow-up, we further explored the performance of the recurrent events joint model under varying sample sizes, length of follow-up time, and strength of association between recurrent events and longitudinal processes through simulations. Based on AIC, the joint models with mixed-effects sub-model of an NB distribution for the parasitemia fitted the data better than the linear mixed-effects model assuming a Gaussian response. For example, we considered a scenario assuming a cohort with a sample size of 200 participants, followed up for 2 years with 10% censoring level, 0.05 correlation level between longitudinal and recurrent processes of 0.05, and frailty term variance for the dependence of within-participant clinical episodes being 0.2. A shared Gamma frailty sub-model with Weibull baseline hazard function is assumed for the recurrent process of clinical malaria episodes. Under this scenario, the joint model with the NB distribution for the parasitemia process yielded a lower AIC value (48,066) compared to that from the Gaussian distribution (549,445). For this reason, subsequent analyses adopted a mixed-effects sub-model with a NB distribution for the parasitemia process.

Tables 2A,B shows log hazard ratio estimates of recurrent clinical malaria for simulated data from the recurrent events joint model obtained by comparing the results under different scenarios. The variables considered included study sample size, length of follow-up time in years, level of censoring, level of correlation between longitudinal and recurrent processes, and frailty term variance for the dependence of within-participant clinical malaria episodes. The joint model consists of a shared Gamma frailty sub-model with Weibull baseline hazard function for the recurrent clinical malaria episodes and a negative binomial mixed-effects sub-model for the parasitemia.

**Table 2A:** Log hazard ratio estimates of recurrent clinical malaria for simulated data under different scenarios for a sample size of study participants; follow-up time in years (*τ*); *c*ensoring level (*C*); recurrent processes (Φ); frailty term (*γ*).

**Variable/Parameter**	**True log hazard**	**Bias**	**SE**	**Bias**	**SE**	**Bias**	**SE**
		***N*** **=** **100**		***N*** **=** **200**		***N*** **=** **400**	
**Scenario 1: C** **=** **10%**, ***τ*** **=** **2**, **Φ** **=** **0.01, var(*γ*)** **=** **0.2**							
Age, per year increase	−0.04	−0.002	0.009	−0.002	0.007	−0.002	0.003
Gender, female	0.2	0.022	0.128	0.007	0.119	0.014	0.041
Season, rainy^*^	0.3	0.029	0.132	0.029	0.110	0.023	0.072
Less LLIN use ^+^	0.3	0.071	0.184	0.043	0.151	0.025	0.069
**Scenario 2: C** **=** **20%**, ***τ*** **=** **2**, **Φ** **=** **0.01, var(*γ*)** **=** **0.2**							
Age, per year increase	−0.04	−0.003	0.006	−0.002	0.005	−0.002	0.003
Gender, female	0.2	0.028	0.106	0.012	0.085	0.012	0.054
Season, rainy^*^	0.3	0.035	0.107	0.035	0.095	0.034	0.051
Less LLIN use^+^	0.3	0.072	0.148	0.042	0.121	0.026	0.085
**Scenario 3: C** **=** **50%**, ***τ*** **=** **2**, **Φ** **=** **0.01, var(*γ*)** **=** **0.2**							
Age, per year increase	−0.04	−0.004	0.013	−0.003	0.005	−0.002	0.005
Gender, female	0.2	0.027	0.157	0.015	0.101	0.021	0.071
Season, rainy^*^	0.3	0.073	0.129	0.046	0.138	0.040	0.058
Less LLIN use ^+^	0.3	0.086	0.204	0.051	0.116	0.042	0.095
**Scenario 4: C** **=** **10%**, ***τ*** **=** **3**, **Φ** **=** **0.01, var(*γ*)** **=** **0.2**							
Age, per year increase	−0.04	−0.002	0.006	−0.001	0.003	−0.001	0.002
Gender, female	0.2	−0.005	0.076	−0.001	0.064	−0.001	0.037
Season, rainy^*^	0.3	0.017	0.088	0.013	0.071	0.009	0.040
Less LLIN use ^+^	0.3	0.036	0.115	0.020	0.087	0.017	0.041
**Scenario 5: C** **=** **10%**, ***τ*** **=** **4**, **Φ** **=** **0.01, var(*γ*)** **=** **0.2**							
Age, per year increase	−0.04	−0.001	0.005	0.000	0.003	−0.001	0.002
Gender, female	0.2	0.018	0.054	−0.001	0.041	−0.001	0.032
Season, rainy^*^	0.3	0.006	0.053	0.010	0.031	0.008	0.030
Less LLIN use ^+^	0.3	0.022	0.072	0.020	0.060	0.017	0.044

* *Rainy season (December–April), reference category is dry season (May–November)*.

+ *Reference category is LLIN use every night in the previous month*.

*SE, standard error; CI, confidence interval*.

**Table 2B:** Log hazard ratio estimates of recurrent clinical malaria for simulated data under different scenarios for a sample size of study participants; correlation level between longitudinal and recurrent processes (Φ); frailty term (*γ*) for fixed *c*ensoring level (*C*).

**Variable/Parameter**	**True log hazard**	**Bias**	**SE**	**Bias**	**SE**	**Bias**	**SE**
		***N*** **=** **100**		***N*** **=** **200**		***N*** **=** **400**	
**Scenario 1: C** **=** **10%**, ***τ*** **=** **2**, **Φ** **=** **0.01, var(*γ*)** **=** **0.2**							
Age, per year increase	−0.04	−0.002	0.009	−0.002	0.007	−0.002	0.003
Gender, female	0.2	0.022	0.128	0.007	0.119	0.014	0.041
Season, rainy^*^	0.3	0.029	0.132	0.029	0.110	0.023	0.072
Less LLIN use ^+^	0.3	0.071	0.184	0.043	0.151	0.025	0.069
**Scenario 6: C** **=** **10%**, ***τ*** **=** **2**, **Φ** **=** **0.01, var(*γ*)** **=** **1.5**							
Age, per year increase	−0.04	−0.003	0.016	−0.002	0.007	0.000	0.003
Gender, female	0.2	0.020	0.184	0.003	0.117	0.001	0.069
Season, rainy^*^	0.3	0.115	0.179	0.043	0.131	0.014	0.072
Less LLIN use^+^	0.3	0.033	0.258	0.006	0.126	0.000	0.103
**Scenario 7: C** **=** **10%**, ***τ*** **=** **2**, **Φ** **=** **0.01, var(*γ*)** **=** **2.5**							
Age, per year increase	−0.04	−0.003	0.016	−0.002	0.007	0.000	0.005
Gender, female	0.2	0.021	0.169	0.014	0.166	0.001	0.087
Season, rainy^*^	0.3	0.038	0.192	0.037	0.155	0.037	0.082
Less LLIN use ^+^	0.3	0.036	0.256	0.016	0.192	0.000	0.116
**Scenario 8: C** **=** **10%**, ***τ* = ** **2**, **Φ = ** **0.5, var(*γ*)** **=** **0.2**							
Age, per year increase	−0.04	−0.002	0.010	−0.002	0.005	−0.001	0.003
Gender, female	0.2	0.018	0.121	0.014	0.112	0.014	0.038
Season, rainy^*^	0.3	0.027	0.130	0.032	0.108	0.022	0.055
Less LLIN use^+^	0.3	0.046	0.170	0.027	0.141	0.012	0.069
**Scenario 9: C** **=** **10%**, ***τ*** **=** **2**, **Φ** **=** **0.8, var(*γ*)** **=** **0.2**							
Age, per year increase	−0.04	−0.004	0.010	−0.003	0.005	−0.001	0.003
Gender, female	0.2	0.021	0.127	0.018	0.100	0.015	0.035
Season, rainy^*^	0.3	0.027	0.131	0.035	0.108	0.021	0.049
Less LLIN use^+^	0.3	0.063	0.170	0.031	0.141	0.013	0.130

* *Rainy season (December–April), reference category is dry season (May–November)*.

+ *Reference category is LLIN use nightly in previous month*.

*SE, standard error; CI, confidence interval*.

The performance of the recurrent events joint model improved with increased study sample size overall as evident from the decreased bias when changing the number of participants from 100, 200 to 400 (Table 2A). The level of censoring denotes the number of known outcomes during the observation time. The increasing level of censoring from 10, 20, to 50% in that order worsened the performance of the joint model as seen from increased bias (Table 2A). The length of study follow-up may determine the number of measurements (information) that a model uses for estimation. The magnitude of bias decreased with increasing follow-up time, as more measurements were available over time. The level of association between recurrent events and longitudinal processes would also determine the performance of the joint model. As shown in Table 2B, the joint model performed best overall with moderate (Φ = 0.5) association between recurrent and longitudinal processes when compared to weak (Φ = 0.01) or strong (Φ = 0.8). Referencing a scenario with low dependence [var(*γ*) = 0.2] of within-participant episodes, there was a decrease in bias for moderately dependent episodes [var(*γ*) = 1.5] but the performance did not improve further when episodes were assumed to be highly dependent [var(*γ*) = 2.5].

## Discussion

Our results demonstrate that jointly modeling recurrent clinical malaria episodes and parasitemia estimates the hazard of clinical malaria with more precision (narrower confidence intervals and smaller standard errors) than a single-event joint model where the subsequent episodes are ignored. The single event joint model gave smaller estimates of hazard ratios, except for age, in most cases with larger standard errors, when compared with the recurrent events joint model. The simulation study shows that if correctly specified, the recurrent events joint model can give parameter estimates with low bias. Exclusion of subsequent episodes by the single event joint model means loss of otherwise valuable information for estimation (42). The recurrent joint model is superior to the traditional approaches in that while the traditional approaches ignore subsequent clinical malaria episodes or repeated parasitemia and hence underestimate the risk of clinical malaria, the recurrent joint model corrects for this bias. Underestimation of parameters may lead to incorrect inferences and wrong conclusions. In this cohort, for example, the season in previous visit seemed not to be associated with the risk of clinical malaria when subsequent episodes were ignored by the single-event model. However, when the recurrent event joint model was used to include all episodes, the rainy season was associated with an increased risk of recurrent clinical malaria. These results support the need for expanded models to utilize all data collected during follow-up to accurately capture the effect of acquired immunity on subsequent clinical malaria episodes due to repeated *P. falciparum* infections.

We found that older age at enrolment was associated with a reduced risk of clinical malaria. Considering participant age as a proxy for the protective effect of clinical malaria, the trend may partially be attributed to acquired immunity over time (20, 21). Being a cohort from a high transmission area, participants are continuously exposed to repeated bites of infected Anopheles mosquitoes (43, 44). The partial immunity developed over time of exposure may not provide complete protection but it reduces the risk that malaria infection will cause the disease (45). Results from this study highlight the need for studies to assess the effect of age on the risk of clinical malaria while accounting for the acquired immunity, and the joint model of recurrent clinical malaria and *P. falciparum* parasitemia is critical. Based on the joint model, the predicted conditional cumulative and marginal non-proportional hazards of clinical malaria show that the expected number of clinical malaria episodes in the cohort increased sharply at the beginning of the follow-up period but later slowed beyond 1 year. Thus, the trend shows fewer clinical malaria episodes in subsequent periods over time in the cohort, consistent with previous studies (21, 46, 47).

In the simulation study, the recurrent events joint model performed differently under varied conditions of study sample size, length of follow-up time, and level of censoring. The performance of the joint model, as measured by decreasing bias, improved with increasing study sample size and length of study follow-up. These results are consistent with previous simulation-based studies in joint modeling (23, 48–50). Thus, model performance improved as more data points were available over time. However, increasing level of censoring worsened the performance, a result in line with other joint modeling reports (50). The joint model performance improved by changing the strength of association between recurrent and longitudinal processes from weak to moderate but there was no further clear improvement when the two processes were assumed to be strongly associated. There was a decrease in the bias of the model by increasing the level of dependence of within-participant episodes from low to moderate, but the performance did not improve further assuming high dependence. Lack of clear trends in model performance with change in the strength of the association or level of dependence of within-participant episodes may be partly attributed to interaction among factors. In this study, factors were varied on a one-by-one basis, and results were compared to the reference scenario. This approach does not allow one to study the effect of interaction between factors. Morris et al. (51) recommend varying factors factorially as this approach may likely be more informative since this allows for the exploration of interactions between factors. However, in this study, the extensive required computational time for the models renders the factorial approach infeasible.

Strengths of this study include a combined approach of using real data to fit the models and a simulation study to investigate how the model would perform under different conditions such as study sample size, follow-up period, and level of censoring. Second, the real data used in this study had limited missingness. Further studies should investigate the role of missing data on the performance of the model under different missing level and mechanisms. Finally, using the joint model, we were able to predict the risk of recurrent clinical episodes. The prediction ability can be crucial when designing malaria interventions. Further studies should focus on model diagnostics of the joint model and utilize tools such as residual plots.

The main limitation of this study was the computational complexity of the likelihood for the joint models, resulting in non-convergence problems of the EM algorithms. Non-convergence is a common problem in the field of joint modeling because of frequent high-dimensional random effects and parameter space. Some examples of joint model simulation studies with documented non-convergence problems include Henderson et al. (5), Ferrer et al. (52), and Xu (53). The computational time further increased with increases in sample size and censoring. The computational time for some simulation models was long, reaching up to 24 hours, using an Intel Core i7 2.5 GHz CPU computer. The non-convergence problem prevented exploration of other simulation scenarios including larger sample sizes and longer study follow-up periods, which might be the practical conditions in most settings.

In conclusion, this study has shown that the recurrent events joint model can provide a way of estimating the risk of recurrent clinical malaria in a cohort where the effect of acquired immunity to malaria disease with aging is captured. Furthermore, the study has demonstrated a decreasing trend in the risk of clinical malaria with aging highlighting the need for expanded analytical methodologies to accurately evaluate such changing effects. Through simulation, this study has shown that, if correctly specified, the recurrent events joint model can estimate the risk of clinical malaria with low bias.

## Data Availability Statement

The original contributions presented in the study are included in the article/Supplementary Materials, further inquiries can be directed to the corresponding author/s.

## Ethics Statement

The data used in this article came from Mfera malaria cohort study which was approved by Institutional Review Boards (IRB)s and Ethics Committees in Malawi and University of Maryland in USA. Ethical clearance for this study was also obtained from Human Research Ethics Committee (HREC)-Medical of University of the Witwatersrand (No M170952). The data was anonymized during analysis. Written informed consent to participate in this study was provided by the participants' legal guardian/next of kin.

## Author Contributions

CS, MM, LK, ML, and TC: study conceptualization and design. ML, DM, and AB: data collection. CS: statistical data analysis and drafting of the manuscript. CS, MM, ML, and TC: led in the interpretation of results. CS, MM, LK, AB, DM, ML, and TC: critical review and approval of the final version of the manuscript. All authors contributed to the article and approved the submitted version.

## Funding

This study was supported by the Fogarty International Center of the National Institutes of Health (Grant number D43TW010075), The Wellcome Trust (Grant number 107754/Z/15/Z), and the National Institute of Allergy and Infectious Diseases (Grant number K24AI114996). Funders have no role in the design of the study and collection, analysis, and interpretation of data and in writing the manuscript.

## Conflict of Interest

The authors declare that the research was conducted in the absence of any commercial or financial relationships that could be construed as a potential conflict of interest.

## Publisher's Note

All claims expressed in this article are solely those of the authors and do not necessarily represent those of their affiliated organizations, or those of the publisher, the editors and the reviewers. Any product that may be evaluated in this article, or claim that may be made by its manufacturer, is not guaranteed or endorsed by the publisher.

## References

[1] Tsiatis AA Davidian M. Joint modeling of Longitudinal and time-to-event data: an overview. Stat Sin. (2004) 14:809–34. Available online at: https://www.jstor.org/stable/24307417

[2] Lawrence Gould A Boye ME Crowther MJ Ibrahim JG Quartey G Micallef S. Joint modeling of survival and longitudinal non-survival data: current methods and issues. Report of the DIA Bayesian joint modeling working group. Stat Med. (2015) 34:2181–95. 10.1002/sim.6141 24634327 PMC4677775

[3] Proust-Lima C Séne M Taylor JMG Jacqmin-Gadda H. Joint latent class models for longitudinal and time-to-event data: a review. Stat Methods Med Res. (2014) 23:74–90. 10.1177/0962280212445839 22517270 PMC5863083

[4] Sudell M Kolamunnage-Dona R Tudur-Smith C. Joint models for longitudinal and time-to-event data: a review of reporting quality with a view to meta-analysis. BMC Med Res Methodol. (2016) 16:168. 10.1186/s12874-016-0272-6 27919221 PMC5139124

[5] Henderson R Diggle P Dobson A. Joint modelling of longitudinal measurements and event time data. Biostatistics. (2000) 1:465–80. 10.1093/biostatistics/1.4.465 12933568

[6] Hickey GL Philipson P Jorgensen A Kolamunnage-Dona R. Joint models of longitudinal and time-to-event data with more than one event time outcome: a review. Int J Biostat. (2018) 14. 10.1515/ijb-2017-0047 29389664

[7] Hickey GL Philipson P Jorgensen A Kolamunnage-Dona R. Joint modelling of time-to-event and multivariate longitudinal outcomes: recent developments and issues. BMC Med Res Methodol. (2016) 16:117. 10.1186/s12874-016-0212-5 27604810 PMC5015261

[8] Rizopoulos D. Joint Models for Longitudinal and Time-to-Event Data: With Applications in R. 1st Edn Biostatistics Series. Boca Raton, FL: Chapman and Hall; CRC Press (2012). 10.1201/b12208

[9] Song X Davidian M Tsiatis AA. A semiparametric likelihood approach to joint modeling of longitudinal and time-to-event data. Biometrics. (2002) 58:742–53. 10.1111/j.0006-341X.2002.00742.x 12495128

[10] Taylor JM Wang Y. Surrogate markers and joint models for longitudinal and survival data. Control Clin Trials. (2002) 23:626–34. 10.1016/S0197-2456(02)00234-9 12505241

[11] Chen Q May RC Ibrahim JG Chu H Cole SR. Joint modeling of longitudinal and survival data with missing and left-censored time-varying covariates. Stat Med. (2014) 33:4560–76. 10.1002/sim.6242 24947785 PMC4189992

[12] Chi YY Ibrahim JG. Joint models for multivariate longitudinal and multivariate survival data. Biometrics. (2006) 62:432–45. 10.1111/j.1541-0420.2005.00448.x 16918907

[13] Brown ER Ibrahim JG. A Bayesian semiparametric joint hierarchical model for longitudinal and survival data. Biometrics. (2003) 59:221–8. 10.1111/1541-0420.00028 12926706

[14] Ibrahim JG Chen M-H Sinha D. Bayesian methods for joint modeling of longitudinal and survival data with applications to cancer vaccine trials. Stat Sin. (2004) 14:863–84.14601770

[15] Billingham L Abrams K. Simultaneous analysis of quality of life and survival data. Stat Methods Med Res. (2002) 11:25–48. 10.1191/0962280202sm269ra 11923992

[16] Sagara I Giorgi R Doumbo OK Piarroux R Gaudart J. Modelling recurrent events: comparison of statistical models with continuous and discontinuous risk intervals on recurrent malaria episodes data. Malar J. (2014) 13:293. 10.1186/1475-2875-13-293 25073652 PMC4132199

[17] Liu L Huang X. Joint analysis of correlated repeated measures and recurrent events processes in the presence of death, with application to a study on acquired immune deficiency syndrome. J R Stat Soc Ser C Appl Stat. (2009) 58:65–81. 10.1111/j.1467-9876.2008.00641.x

[18] Marsh K Kinyanjui S. Immune effector mechanisms in malaria. Parasite Immunol. (2006) 28:51–60. 10.1111/j.1365-3024.2006.00808.x 16438676

[19] Moorthy VS Reed Z Smith PG. Clinical trials to estimate the efficacy of preventive interventions against malaria in paediatric populations: a methodological review. Malaria J. (2009). 8:23. 10.1186/1475-2875-8-23 19208236 PMC2646744

[20] Doolan DL Dobaño C Baird JK. Acquired immunity to malaria. Clin Microbiol Rev. (2009) 22:13–36. 10.1128/CMR.00025-08 19136431 PMC2620631

[21] Biswas S Seth RK Tyagi PK Sharma SK Dash AP. Naturally acquired immunity and reduced susceptibility to falciparum malaria in two subpopulations of endemic Eastern India. Scand J Immunol. (2008) 67:177–84. 10.1111/j.1365-3083.2007.02047.x 18086262

[22] Lin H Turnbull BW McCulloch CE Slate EH. Latent class models for joint analysis of longitudinal biomarker and event process data: application to longitudinal prostate-specific antigen readings and prostate cancer. J Am Stat Assoc. (2002) 97:53–65. 10.1198/016214502753479220

[23] Han J Slate EH Peña EA. Parametric latent class joint model for a longitudinal biomarker and recurrent events. Stat Med. (2007) 26:5285–302. 10.1002/sim.2915 17542002 PMC4066416

[24] Rizopoulos D Verbeke G Molenberghs G. Shared parameter models under random effects misspecification. Biometrika. (2008) 95:63–74. 10.1093/biomet/asm087

[25] Clayton DG. A model for association in bivariate life tables and its application in epidemiological studies of familial tendency in chronic disease incidence. Biometrika. (1978) 65:141–51. 10.1093/biomet/65.1.141

[26] Clayton D. Some approaches to the analysis of recurrent event data. Stat Methods Med Res. (1994) 3:244–62. 10.1177/096228029400300304 7820294

[27] Shen Y Huang H Guan Y. A conditional estimating equation approach for recurrent event data with additional longitudinal information. Stat Med. (2016) 35:4306–19. 10.1002/sim.7001 27241902

[28] Buchwald AG Sorkin JD Sixpence A Chimenya M Damson M Wilson ML. Association between age and *Plasmodium falciparum* infection dynamics. Am J Epidemiol. (2019) 188:169–76. 10.1093/aje/kwy213 30252032 PMC6321803

[29] Buchwald AG Sixpence A Chimenya M Damson M Sorkin JD Wilson ML. Clinical implications of asymptomatic *Plasmodium falciparum* infections in Malawi. Clin Infect Dis. (2019) 68:106–12. 10.1093/cid/ciy427 29788054 PMC6293006

[30] Earland D Buchwald AG Sixpence A Chimenya M Damson M Seydel K. Impact of multiplicity of *Plasmodium falciparum* infection on clinical disease in Malawi. Am J Trop Med Hyg. (2019) 101:412–5. 10.4269/ajtmh.19-0093 31219007 PMC6685583

[31] Stanley CC Kazembe LN Buchwald AG Mukaka M Mathanga DP Hudgens MG. Joint modelling of time-to-clinical malaria and parasite count in a cohort in an endemic area. J Med Stat Informatics. (2019) 7:1. 10.7243/2053-7662-7-1 31245015 PMC6594707

[32] World Health Organization (WHO). Epidemiological profile of malaria in Malawi in 2015 [Internet]. (2017) Available online at: http://www.who.int/malaria/publications/country-profiles/profile_mwi_en.pdf?ua=1 (Accessed July 11, 2017).

[33] Bennett A Kazembe L Mathanga DP Kinyoki D Ali D Snow RW. Mapping malaria transmission intensity in Malawi, 2000-2010. Am J Trop Med Hyg. (2013) 89:840–9. 10.4269/ajtmh.13-0028 24062477 PMC3820324

[34] Walldorf JA Cohee LM Coalson JE Bauleni A Nkanaunena K Kapito-Tembo A. School-age children are a reservoir of malaria infection in Malawi. PLoS ONE. (2015) 10:e134061. 10.1371/journal.pone.0134061 26207758 PMC4514805

[35] Bartoloni A Zammarchi L. Clinical aspects of uncomplicated and severe malaria. Mediterr J Hematol Infect Dis. (2012) 4:e2012026. 10.4084/mjhid.2012.026 22708041 PMC3375727

[36] Zhang X Mallick H Tang Z Zhang L Cui X Benson AK. Negative binomial mixed models for analyzing microbiome count data. BMC Bioinformatics. (2017) 18:4. 10.1186/s12859-016-1441-7 28049409 PMC5209949

[37] Pickles A Crouchley R. A comparison of frailty models for multivariate survival data. Stat Med. (1995) 14:1447–61. 10.1002/sim.4780141305 7481183

[38] Hsieh F Tseng YK Wang JL. Joint modeling of survival and longitudinal data: likelihood approach revisited. Biometrics. (2006) 62:1037–43. 10.1111/j.1541-0420.2006.00570.x 17156277

[39] Wang MC Qin J Chiang CT. Analyzing recurrent event data with informative censoring. J Am Stat Assoc. (2001) 96:455. 10.1198/016214501753209031 24204084 PMC3818252

[40] R Core Team. R Core Team. R: A Language and Environment for Statistical Computing. Vienna: R Foundation for Statistical Computing (2017).

[41] StataCorp. Stata Statistical Software: Release 15. College Station, TX: StataCorp LP (2017).

[42] Huang X Liu L. A joint frailty model for survival and gap times between recurrent events. Biometrics. (2007) 63:389–97. 10.1111/j.1541-0420.2006.00719.x 17688491

[43] Guelbéogo WM Gonçalves BP Grignard L Bradley J Serme SS Hellewell J. Variation in natural exposure to anopheles mosquitoes and its effects on malaria transmission. Elife. (2018) 7:e32625. 10.7554/eLife.32625 29357976 PMC5780040

[44] Bousema T Sutherland CJ Churcher TS Mulder B Gouagna LC Riley EM. Human immune responses that reduce the transmission of *Plasmodium falciparum* in African populations. Int J Parasitol. (2011) 41:293–300. 10.1016/j.ijpara.2010.09.008 20974145 PMC3052432

[45] Cohen S McGregor IA Carrington S. Gamma-globulin and acquired immunity to human malaria. Nature. (1961) 192:733–7. 10.1038/192733a0 13880318

[46] Gatton ML Cheng Q. Modeling the development of acquired clinical immunity to *Plasmodium falciparum* malaria. Infect Immun. (2004) 72:6538–45. 10.1128/IAI.72.11.6538-6545.2004 15501785 PMC523055

[47] Aponte JJ Menendez C Schellenberg D Kahigwa E Mshinda H Vountasou P. Age interactions in the development of naturally acquired immunity to *Plasmodium falciparum* and its clinical presentation. PLoS Med. (2007) 4:e242. 10.1371/journal.pmed.0040242 17676985 PMC1950208

[48] Arisido MW Antolini L Bernasconi DP Valsecchi MG Rebora P. Joint model robustness compared with the time-varying covariate Cox model to evaluate the association between a longitudinal marker and a time-to-event endpoint. BMC Med Res Methodol. (2019) 19:222. 10.1186/s12874-019-0873-y 31795933 PMC6888912

[49] Roustaei N Ayatollahi SMT Zare N. A proposed approach for joint modeling of the longitudinal and time-to-event data in heterogeneous populations: an application to HIV/AIDS's disease. Biomed Res Int. (2018) 2018:7409284. 10.1155/2018/7409284 29546067 PMC5818956

[50] Efendi A Molenberghs G Njagi EN Dendale P. A joint model for longitudinal continuous and time-to-event outcomes with direct marginal interpretation. Biometr J. (2013) 55:572–88. 10.1002/bimj.201200159 23606452

[51] Morris TP White IR Crowther MJ. Using simulation studies to evaluate statistical methods. Stat Med. (2019) 38:2074–102. 10.1002/sim.8086 30652356 PMC6492164

[52] Ferrer L Rondeau V Dignam J Pickles T Jacqmin-Gadda H Proust-Lima C. Joint modelling of longitudinal and multi-state processes: application to clinical progressions in prostate cancer. Stat Med. (2016) 35:3933–48. 10.1002/sim.6972 27090611 PMC5012926

[53] Xu C Chinchilli VM Wang M. Joint modeling of recurrent events and a terminal event adjusted for zero inflation and a matched design. Stat Med. (2018) 37:2771–86. 10.1002/sim.7682 29682772 PMC7249437

