# Supplementary material for: Analysis of Recurrent Times-to-Clinical Malaria Episodes and Plasmodium falciparum Parasitemia: A Joint Modeling Approach Applied to a Cohort Data
Source: Front Epidemiol. 2022 Jul 8;2:924783. doi: 10.3389/fepid.2022.924783 (PMC10911024; doi:10.3389/fepid.2022.924783)
Supplement: Supplementary file 1 [file Table_1.DOCX]

| **STable 2: Baseline characteristics of the cohort data** | | | | |
| --- | --- | --- | --- | --- |
| Variable | |  |  | Total (n=120) |
| Gender, female, n (%) | |  |  | 69 (57.4) |
| Age, n (%) | |  |  |  |
|  | < 5 years |  |  | 34 (28.3) |
|  | 5-15 years |  |  | 51 (42.5) |
|  | >15 years |  |  | 35 (29.2) |
| Weight (kg), median (IQR) | |  |  | 21.5 (15.0 - 46.0) |
| Height (cm), median (IQR) | |  |  | 119.5 (103.0 - 151.8) |
| Temperature (℃), median (IQR) | |  |  | 36.7 (36.2 - 38.6) |
| Respiratory rate (breaths/minute), median (IQR) | |  |  | 28 (22 - 36) |
| Heart rate (beats/minute), median (IQR) | |  |  | 112 (92 - 139) |
| Haemoglobin (g/dl), median (IQR) | |  |  | 11.5 (10.2 - 12.4) |
| Parasite count (number of parasites/µL), median (IQR) | |  |  | 11060 (840 - 54000) |
| Cough, n (%) | |  |  | 15 (12.5) |
| Musculoskeletal pain, n (%) | |  |  | 40 (33.3) |
| Headache, n (%) | |  |  | 36 (30.0) |
| Vomiting, n (%) | |  |  | 32 (26.7) |
| Abdominal pain, n (%) | |  |  | 15 (12.5) |
| Bed net use previous month*, n (%) | |  |  |  |
|  | Every night |  |  | 48 (44.9) |
|  | Most nights (> half) |  |  | 13 (12.1) |
|  | Some nights (< half) |  |  | 8 (7.5) |
|  | No nights |  |  | 38 (35.5) |
| Season enrolled, n (%) | | | | |
|  | Dry: May - November |  |  | 91 (75.8) |
|  | Rainy: December - April |  |  | 29 (24.2) |
|  | *not adding up to column total due to missing | |  |  |
