# Supplementary material for: Analysis of Recurrent Times-to-Clinical Malaria Episodes and Plasmodium falciparum Parasitemia: A Joint Modeling Approach Applied to a Cohort Data
Source: Front Epidemiol. 2022 Jul 8;2:924783. doi: 10.3389/fepid.2022.924783 (PMC10911024; doi:10.3389/fepid.2022.924783)
Supplement: Supplementary file 2 [file Table_2.DOCX]

Supporting table 1: Data structure showing three hypothetical participants for the analysis of time-to-recurrent clinical malaria episodes

| **ID** | **Start** | **Stop** | **Time** | **Episode** | **Order** | **Age** | **Gender** | **Season** |
| --- | --- | --- | --- | --- | --- | --- | --- | --- |
|  |  | **(Days)** |  |  |  |  |  |  |
| 1 | 0 | 27 | 27 | 1 | 1 | 5 | Male | Rainy |
| 1 | 41 | 56 | 15 | 1 | 2 | 5 | Male | Rainy |
| … | … | … | … | … | … | … | … | … |
| 1 | 358 | 386 | 28 | 0 | 10 | 5 | Male | Dry |
| 2 | 0 | 31 | 31 | 1­­ | 1 | 3 | Female | Dry |
| 2 | 45 | 62 | 17 | 1 | 2 | 3 | Female | Dry |
| 2 | 76 | 98 | 22 | 1 | 3 | 3 | Female | Dry |
| 3 | 0 | 34 | 34 | 1 | 1 | 36 | Male | Rainy |
| 3 | 48 | 81 | 33 | 0 | 2 | 36 | Male | Rainy |

Key:

ID: study participant identification number; Start: start time in days of the interval; End: end time in days at which the event occurs or the time of censoring; Time: the number of days at risk as a difference Start from End; Episode-whether the clinical malaria episode occurred (yes = 1, no = 0); Order: order of the episodes for a participant during follow-up; Age- participant’s age at enrolment in years; Gender: participants’ gender (Female, Male); Season: Season during visit (Dry, Rainy).
